# Applying next generation sequencing with microdroplet PCR to determine the disease-causing mutations in retinal dystrophies

**DOI:** 10.1186/s12886-017-0549-5

**Published:** 2017-08-24

**Authors:** Xinjing Wang, Wadih M. Zein, Leera D’Souza, Chimere Roberson, Keith Wetherby, Hong He, Angela Villarta, Amy Turriff, Kory R. Johnson, Yang C. Fann

**Affiliations:** 1 Ophthalmic Genetics and Visual Function Branch, National Eye Institute, National Institutes of Health, 10D43, 10 Center Drive, Bethesda, MD 20892 USA; 2 Intramural IT and Bioinformatics Program, National Institute of Neurological Disorders and Stroke, National Institutes of Health, Bethesda, MD USA

**Keywords:** Retinal, Microdroplet PCR, Next-generation-sequencing, Mutation screening

## Abstract

**Background:**

Inherited Retinal dystrophy (IRD) is a broad group of inherited retinal disorders with heterogeneous genotypes and phenotypes. Next generation sequencing (NGS) methods have been broadly applied for analyzing patients with IRD. Here we report a novel approach to enrich the target gene panel by microdroplet PCR.

**Methods:**

This assay involved a primer library which targeted 3071 amplicons from 2078 exons comprised of 184 genes involved in retinal function and/or retinal development. We amplified the target regions using the RainDance target enrichment PCR method and sequenced the products using the MiSeq NGS platform.

**Results:**

In this study, we analyzed 82 samples from 67 families with IRD. Bioinformatics analysis indicated that this procedure was able to reach 99% coverage of target sequences with an average sequence depth of reads at 119×. The variants detected by this study were filtered, validated, and prioritized by pathogenicity analysis. Genotypes and phenotypes were correlated by determining a consistent relationship in 38 propands (56.7%). Pathogenic variants in genes related to retinal function were found in another 11 probands (16.4%), but the clinical correlations showed inconsistencies and insufficiencies in these patients.

**Conclusions:**

The application of NGS in IRD clinical molecular diagnosis provides a powerful approach to exploring the etiology and pathology in patients. It is important for the clinical laboratory to interpret the molecular findings in the context of patient clinical presentations because accurate interpretation of pathogenic variants is critical for delivering solid clinical molecular diagnosis to clinicians and patients and improving the standard care of patients.

**Electronic supplementary material:**

The online version of this article (doi:10.1186/s12886-017-0549-5) contains supplementary material, which is available to authorized users.

## Background

Inherited Retinal dystrophy (IRD) is a clinically and genetically heterogeneous group of disorders that affects approximately 1 in 2000 individuals worldwide [1]. Clinically IRD can be classified according to the clinical phenotype and course of the disease, as well as the underlying inheritance. However, sporadic retinal dystrophies, such as Retinitis Pigmentosa (RP), Leber Congenital Amaurosis (LCA), Cone and Cone-Rod dystrophy (CRD), Macular Dystrophy (MD), and Congenital Stationary Night Blindness (CSNB) can present difficulties in the clinical differential diagnosis. The heterogeneity of some syndromic conditions with retinal abnormalities (i.e., Usher syndrome, Bardet-Biedl syndrome, and Nephronophthisis) could add more complexity because these diseases are caused by multiple underlying gene defects [2, 3]. In molecular pathology, IRD may result from mutations in a variety of genes and may show different inheritance patterns, including autosomal dominant, autosomal recessive, X-linked, and mitochondrial inheritance [3, 4]. Additionally, mutations within the same gene may be associated with different phenotypes [2, 5]. Although monogenic forms have been reported in most families, some digenic forms have also been identified [2, 3].

Retinitis pigmentosa is the most frequent type of IRD and it is also highly genetic heterogeneous. More than 50 genes have been identified responsible for RP with similar clinical presentations. Typically, it presents night blindness and peripheral visual field decreasing. As part of progression in RP, nigh blindness will eventually become blindness. On the other hand, the night blindness is also a typical presentation of CSDB. While CSDB can be distinguished by electroretinogram (ERG), more than 10 genes have been identified responsible for CSDB and a portion of CSDB patients were found no mutation in the known CSDB genes. LCA, CRD, MD, and Occult Macular Dystrophy (OCMD) can be differentiated clinically in general, but the molecular pathology of malfunction in cone photoreceptors cannot be established without identifying the disease-causing gene in each patient.

To date, more than 200 genes have been identified for different retinal diseases by a variety of methods (http://www.sph.uth.tmc.edu/retnet/sum-dis.htm). Significant progress has been made in determining the molecular causes of IRD, but much more work remains to be done.

Genetic testing for IRD can identify causative mutations but requires sequencing of many individual candidate genes. Next generation sequencing (NGS) has been broadly applied for analyzing patients with IRD. NGS enables rapid and cost-effective parallel sequencing of a large panel of disease genes. It offers an ideal model in a clinical diagnostic setting. Many studies investigating sequence-capture technology with selected gene targets for enrichment have been published [6–10]. A significantly higher rate of molecular diagnosis (as compared to Sanger sequencing potential candidate genes) of well above 50% was also achieved. Furthermore, NGS can be adapted to include other retinal diseases without significantly increasing the cost. In this study, we performed a comprehensive molecular screening of patients by NGS using Illumina MiSeq platform and target enrichment using a customized primer library based microdroplet PCR. We accomplished targeted gene panel sequencing of 184 genes in 74 patients with different inheritance patterns and clinical diagnoses including Choroidermia (CHM), CRD, CSNB, LCA, MD, OCMD, and RP. Careful clinical evaluation and follow-up led to the more precise clinical diagnoses and extensive phenotyping in these RD families. Collectively, this study underscores the importance of combining comprehensive molecular screening and clinical information to accurately diagnose diverse retinal disorders.

## Methods

### Subjects and clinical investigation

A set of 82 genomic DNA samples was included in this study. This cohort includes 74 patients with clinical diagnosis of Choroidermia (CHM), CRD, CSNB, LCA, MD, Occult Macular Dystrophy (OCMD), and RP from the NEI Ophthalmic Genetics Clinic or tested in the NEI DNA Diagnostic Laboratory. Clinical evaluations included measurement of best-corrected visual acuity, visual field assessment (either kinetic or static perimetry depending on patient presentation), fundus biomicroscopy and indirect ophthalmoscopy. Optical coherence tomography (Stratus OCT 3 or Cirrus, Carl Zeiss Meditec, Inc., Dublin, CA) was performed. Color and autofluorescence fundus imaging was obtained. International Society for Clinical Electrophysiology of Vision (ISCEV) standard electroretinography responses were obtained using a Burian-Allen contact lens electrode and an LKC-2000 system (LKC Technologies, Gaithersburg, MD). Systemic examinations were performed whenever necessary. Genomic DNA was isolated from peripheral leukocytes using the Gentra Puregene Blood Kit (Qiagen Inc., Valencia, CA) in accordance with the manufacturer’s protocol. Whenever available, a blood sample from affected and unaffected family members was collected for co-segregation analysis. Pedigrees were constructed based on patient interviews. This study was reviewed and approved by the Combined Neuroscience Institutional Review Board of the National Institutes of Health and informed consent was obtained from each participant as adhering to tenets of the Declarations of Helsinki.

### Molecular genetic analysis

#### Design of the microdroplet-based PCR primer library

A panel of 184 RD genes responsible for retinal related diseases was provided to RainDance Technologies Inc. (Billerica, MA) for the design of a primer library in 2011, using their custom primer design pipeline based on the Primer3 algorithm (Additional file 1: Table S1) (http://frodo.wi.mit.edu/primer3). A total of 3071 primer pairs were designed to target the 2078 coding exons of the184 RD genes (Additional file 2: Table S2). RD genes included in this study were previously associated with RD in the literature, RetNet database (http://www.sph.uth.tmc.edu/retnet/sum-dis.htm) and the OMIM database [11] (http://www.ncbi.nlm.nih.gov/omim).

#### RainDance target enrichment and NGS sequencing

Before amplification, the samples were fragmented to 5 kb by shearing the genomic DNA with the Covaris M220 instrument (Covaris, Woburn, MA) and the preparation for amplification was following the manufacturer’s recommended protocol [11]. The samples (50 ng) then entered the sample preparation protocol (NEXTflex PCR-Free DNA Sequencing Kit and NEXTflex PCR-Free barcode 1, Illumina®-Compatible, BIOO Scientific, Austin, Texas), followed by 150 bp pair-end sequencing on a MiSeq instrument (Illumina, San Diego, CA). Samples in each batch were indexed using 12 different index tags (Nextera, Epicentre Biotechnologies, Madison, WI).

#### Variant detection

Reference gene sequences were annotated with known single-nucleotide polymorphisms (SNPs) from the NCBI dbSNP database build 130, 1000 Genomes database build 201,105 and 201,011 and mutations from the Human Gene Mutation Database (HGMD) (http://www.hgmd.cf.ac.uk/) or Leiden Open Variation Database (LOVD) (http://www.lovd.nl/3.0) or reported in the literature. Other variant reference databases used were following: HapMap variants database (Ensembl), Clinical Variants in dbSNP database (NCBI), 1000 genomes database (Ensembl), Chromosome bands ideogram (UCSC), dbSNP common variants (UCSC), Genomic Annotations (Ensembl), Cosmic Noncoding Variants & Coding Mutations (http://cancer.sanger.ac.uk/cosmic).

The sequencing reads were aligned to the reference sequence from NCBI (hg19 Build 37) and analyzed using the CLC Bio Genomics Workbench software™ (QIAGEN). Data was analyzed by using the Probabilistic Variant Detection Tool provided by the Genomics Workbench. Mutation calls were made under robust and stringent sequencing criteria set by the CLC Genomics Workbench with a probability call of 100 in order to delete any false mutation observations. Identified sequence variants were annotated according to the guidelines published by the Human Genome Variation Society (HGVS).

#### Determination of pathogenic variants

The potential pathogenicity of sequence variants was assessed as follows. First, reported SNP with allele frequency above 2% were excluded. Second, potential damaging nucleotide substitution variants defined as nonsense, missense, silent with predicted splicing effects, intronic splice-site variants and any novel variants in the target regions were included. Damaging indels located in the coding regions of the gene were also included. The variants that met the above criteria were selected for downstream analysis as candidate alleles. The pathogenic effect of every candidate allele on gene expression and protein function was assessed in silico using the Alamut Visual software version 2.7 (Alamut, Rouen, France) (www.interactivebiosoftware.com). The pathogenicity assessment includes PolyPhen-2 (Polymorphism Phenotyping) (http://genetics.bwh.harvard.edu/pph2/), SIFT (http://sift.jcvi.org/), and Mutation Taster (http://www.mutationtaster.org/). Synonymous and intronic sequence variants were assessed for potential deleterious effects upon messenger RNA splicing using the Human Splicing Finder V.2.4 tool (http://www.umd.be/HSF/). The Alamut analysis generated a report per variant for the final interpretation.

#### Validation of mutations by Sanger sequencing and segregation

All pathogenic variants identified by NGS after variant filtering were validated by Sanger sequencing as previously described [12]. Reference DNA sequences were obtained from the NCBI. Primer3 (http://frodo.wi.mit.edu/primer3/) was used to design new primers wherever applicable otherwise primers that were used in the RainDance Inc. designed primer library were used in the study.

## Results

### Patients

A total of 82 samples including 74 patients and 4 unaffected family members from 67 unrelated RD families were included in this study. In addition, 3 mutation positive samples that were previously studied in this lab and one DNA control sample acquired from Coriell Repository were included in this study [12]. Clinical examinations were performed in the NEI Ophthalmic Genetics Clinic and patients were clinically diagnosed with CHM, CRD, CSNB, LCA, MD, OCMD, and RP. Patient samples referred from outside clinics were previously tested in the NEI DNA Diagnostic Laboratory.

### Evaluation and validation of the novel RD gene panel

**Fig. 1:**
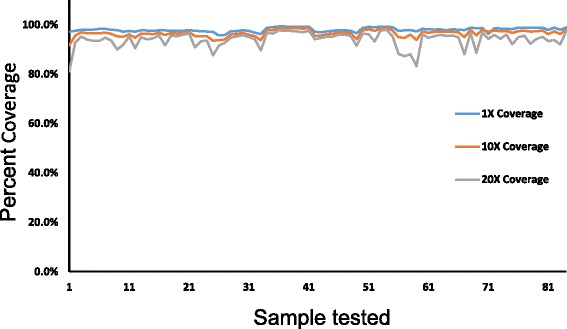
Target coverage analysis. Sequence percent coverage per sample was analyzed. The percent coverage at 1X, 10X, and 20X depths was plotted

NGS sequencing data were processed and analyzed through a bioinformatic pipeline by the Genomic Workbench and the Alamut Visual. An average of 1.14 million reads was generated per sample, an average of 59.5% of reads mapped to the targeted regions. Bioinformatic analysis indicated that this procedure was able to reach 99% of coverage on target sequences with averages of 97% at 1X, 95% at 10X, and 90% at 20X. The average read depth was 119X. The read depth per sample was evaluated at 1X, 10X, and 20X and summarized in the Fig. 1.

### Variant filtering and annotation

On average, our bioinformatic pipeline generated 500–600 variants per sample. After filtering out common polymorphisms with a frequency > 2% in any of the variant databases queried, including 1000 Genome build 201,105 and 201,011 (Genomes Project 2010) and dbSNP130 (NCBI dbSNP database build 130), an average around 30–60 variants remained per sample. The remaining variants were further filtered based on our secondary functional selection as stopgain or stoploss variants, missense variants, synonymous variants with predicted splicing effects, and intronic variants within the 25 bp of exon flanking regions with predicted splicing effects. We had an average of 10 candidate variants per sample for further Alamut pathogenicity analysis and reporting.

### Identification of known and novel mutations

Sanger sequencing was used to analyze whether a variation segregated with the disease phenotype in the corresponding family (whenever additional family members were available). Using this approach, we confirmed 63 previously determined pathogenic variants and 28 novel variants predicted as pathogenic or likely pathogenic in our analyses (Additional file 3: Table S3).

### Clinical correlations

The clinical correlation was performed after mutations and variations were determined and verified by Sanger sequencing. We found that phenotype-genotype relationship could be established in 38 of the 67 probands (56.7%) and the additional affected family members were confirmed with the familial mutations (Table 1). We included 6 samples from a large Occult Macular Dystrophy (OCMD) family. In the three affected members, we confirmed the known mutation, p.R45W in the *RP1L1* gene and excluded the potential of additional pathogenic variants in other retinal disease related genes. We also confirmed the mutation carrying status in the other three unaffected family members.

**Table 1:** Clinical correlated pathogenic variants

Sample ID	Clincial Diagnosis	Family Inheritance	Candidate Gene(s)	Transcript	Nucleotide Change	Amino Acid Change	Genotypes (Sanger confirmed)	HGMD or dbSNP IDs	References (or in silico Analysis)	
RD13–01	CRD	Autosomal dominant	*GUCA1A*	NM_000409.3	c.296A > G	p.Y99C	Heterozygous	CM980960		[26]
RD1–07	RP	Autosomal dominant	*PRPF31*	NM_015629.3	c.220C > T	p.Q74*	Heterozygous	CM063073		[27]
RD20–06	CRD	Autosomal dominant	*PRPH2*	NM_000322.4	c.514C > T	p.R172W	Heterozygous	CM930639		[28]
RD20–07	CRD	Autosomal dominant	*PRPH2*	NM_000322.4	c.514C > T	p.R172W	Heterozygous	CM930639		[28]
RD2–01	RD	Autosomal dominant	*RGR*	NM_001012720.1	c.824dupG	p.I276N*77	Heterozygous	CI993291		[16]
RD11–03	RP	Autosomal dominant	*RHO*	NM_000539.3	c.574dup	p.Y192Lfs*139	Heterozygous	novel	(predicted as pathogenic)	
RD14–07	RP	Autosomal dominant	*TOPORS*	NM_005802.4	c.2474dupA	p.Y825*	Heterozygous	CSI075704		[29]
RD10–02	OCMD	Autosomal dominant	*RP1L1*	NM_178857.5	c.133C > T	p.R45W	Heterozygous	CM105618		[20]
RD5–08	RP	Autosomal recessive	*CERKL*	NM_001030311.2	c.1045_1046del	p.M349Vfs*20	Heterozygous	Novel (predicted as pathogenic)		
					c.316C > T	p.R106C	Heterozygous	Novel	(predicted as likely pathogenic)	
RD14–08	RP	Autosomal recessive	*USH2A*	NM_206933.2	c.2276G > T	p.C759F	Heterozygous	CM001372		[30]
					c.4108G > C	p.V1370 L	Heterozygous	novel	(predicted as pathogenic)	
RD20–08	RP	Autosomal recessive	*USH2A*	NM_206933.2	c.2299delG	p.E766Sfs*21	Heterozygous	CD982997		[9]
					c.2276G > T	p.C759F	Heterozygous	CM001372		[30]
RD20–05	CSNB	X-linked	*CACNA1F*	NM_005183.3	c.2576 + 1G > A	IVS20 + 1G > A	Hemizygous	novel	(predicted as pathogenic)	
RD6–08	CHM	X-linked	*CHM*	NM_000390.2	c.49 + 2dupT	IVS1 + 2dupT	Hemizygous	CI137323		[31]
RD12–02	RP	X-linked	*RPGR*	NM_000328.2	c.1088_1089delinsA	p.V363Dfs*18	Hemizygous	novel	(predicted as pathogenic)	
RD4–04	RP	Sporadic	*ABCA4*	NM_000350.2	c.1804C > T	p.R602W	Homozygous	CM990025		[32]
RD6–01	RP	Sporadic	*BBS1*	NM_024649.4	c.1169 T > G	p.M390R	Homozygous	CM021489		[33]
RD12–06	RP	Sporadic	*CERKL*	NM_001030311.2	c.481 + 2 T > G	IVS2 + 2 T > G	Homozygous	CS140556		[7]
RD5–05	RP	Sporadic	*CRB1*	NM_201253.2	c.3983C > A	p.A1328D	Heterozygous	rs762975680	(predicted as likely pathogenic)	
RD4–06	RP	Sporadic	*EYS*	NM_001142800.1	c.2259 + 1G > A	IVS14 + 1G > A	Heterozygous	CS150721		[34]
					c.6137G > A	p.W2046*	Heterozygous	novel	(predicted as pathogenic)	
RD6–07	RP	Sporadic	*EYS*	NM_001142800.1	c.8473_8474insT	p.V2804 fs	Heterozygous	novel	(predicted as pathogenic)	
					c.1153 T > G	p.C385G	Heterozygous	Novel	*(predicted as unlikely pathogenic)*	
RD12–05	RP	Sporadic	*EYS*	NM_001142800.1	c.6416G > A	p.C2139Y	Heterozygous	CM102730		[25]
					c.7868G > A	p.G2623E	Heterozygous	novel	(predicted as likely pathogenic)	
RD15–04	RP	Sporadic	*EYS*	NM_001142800.1	c.2975G > T	p.C992F	Homozygous	rs566917467	(predicted as pathogenic)	
RD13–05	CRD	Sporadic	*GUCY2D*	NM_000180.3	c.2375C > T	p.P792L	Heterozygous	rs763774686	(predicted as pathogenic)	
RD11–08	RP	Sporadic	*IMPDH1*	NM_000883.3	c.931G > A	p.D311N	Heterozygous	CM020283		[35]
RD15–03	RP	Sporadic	*IMPDH1*	NM_000883.3	c.931G > A	p.D311N	Heterozygous	CM020283		[35]
RD5–04	RP	Sporadic	*RHO*	NM_000539.3	c.68C > A	p.P23H	Heterozygous	CM900197		[36]
RD13–02	RP	Sporadic	*RHO*	NM_000539.3	c.68C > A	p.P23H	Heterozygous	CM900197		[36]
RD12–08	RP	Sporadic	*RHO*	NM_000539.3	c.561 T > G	p.C187W	Heterozygous	novel	(predicted as pathogenic)	
RD12–07	RP	Sporadic	*RHO*	NM_000539.3	c.936 + 1G > T	IVS4 + 1G > T	Heterozygous	CS920776		[37]
RD14–06	RD	Sporadic	*PRPH2*	NM_000322.4	c.514C > T	p.R172W	Heterozygous	CM930639		[28]
RD12–03	RP/LCA	Sporadic	*RPE65*	NM_000329.2	c.886dup	p.R296Kfs*7	Homozygous	CI107001		[38]
RD12–01	RP	Sporadic	*RPGR*	NM_000328.2	c.197A > G	p.Q66R	Hemizygous	novel	(predicted as pathogenic)	
RD6–05	MD	Sporadic	*TIMP3*	NM_000362.4	c.29 T > A	p.L10H	Heterozygous	Novel	(predicted as pathogenic)	
RD20–03	CSNB	Sporadic	*TRPM1*	NM_002420.5	c.1197G > A	p.P421=	Heterozygous	CS097758		[25]
					c.215A > G	p.Y72C	Heterozygous	CM097760		[25]
RD6–04	CSNB	Sporadic	*TRPM1*	NM_002420.5	c.2947_2948delGCinsAT	p.A983I	Heterozygous	Novel	(predicted as pathogenic)	
					c.3125 T > G	p.L1042R	Heterozygous	Novel	(predicted as likely pathogenic)	
RD5–07	RP	Sporadic	*USH2A*	NM_206933.2	c.2276G > T	p.C759F	Homozygous	CM001372		[30]
RD14–02	RP	sporadic	*USH2A*	NM_206933.2	c.11411del	p.P3804Lfs*13	Heterozygous	CD149996		[39]
					c.8431C > A	p.P2811T	Heterozygous	rs111033529	(predicted as likely pathogenic)	
RD11–04	RP	Sporadic	*USH2A*	NM_206933.2	c.13335_13347delinsCTTG	p.E4445_S4449delinsDL	Heterozygous	CX104126		[40]
*Additional affected family members*										
RD14–04	CRD	Autosomal dominant	*GUCA1A*	NM_000409.3	c.296A > G	p.Y99C	Heterozygous	CM980960		[26]
RD2–02	RD	Autosomal dominant	*RGR*	NM_001012720.1	c.824dupG	p.I276N*77	Heterozygous	CI993291		[16]
RD10–02	OCMD	Autosomal dominant	*RP1L1*	NM_178857.5	c.133C > T	p.R45W	Heterozygous	CM105618		[20]
RD10–06	OCMD	Autosomal dominant	*RP1L1*	NM_178857.5	c.133C > T	p.R45W	Heterozygous	CM105618		[20]
RD15–02	RP	Autosomal recessive	*CERKL*	NM_001030311.2	c.1045_1046del	p.M349Vfs*20	Heterozygous	Novel	(predicted as pathogenic)	
					c.316C > T	p.R106C	Heterozygous	Novel	(predicted as likely pathogenic)	
RD20–02	RP	Autosomal recessive	*USH2A*	NM_206933.2	c.2299delG	p.E766Sfs*21	Heterozygous	CD982997		[9]
					c.2276G > T	p.C759F	Heterozygous	CM001372		[30]

### Inconclusive genotypes

**Fig. 2:**
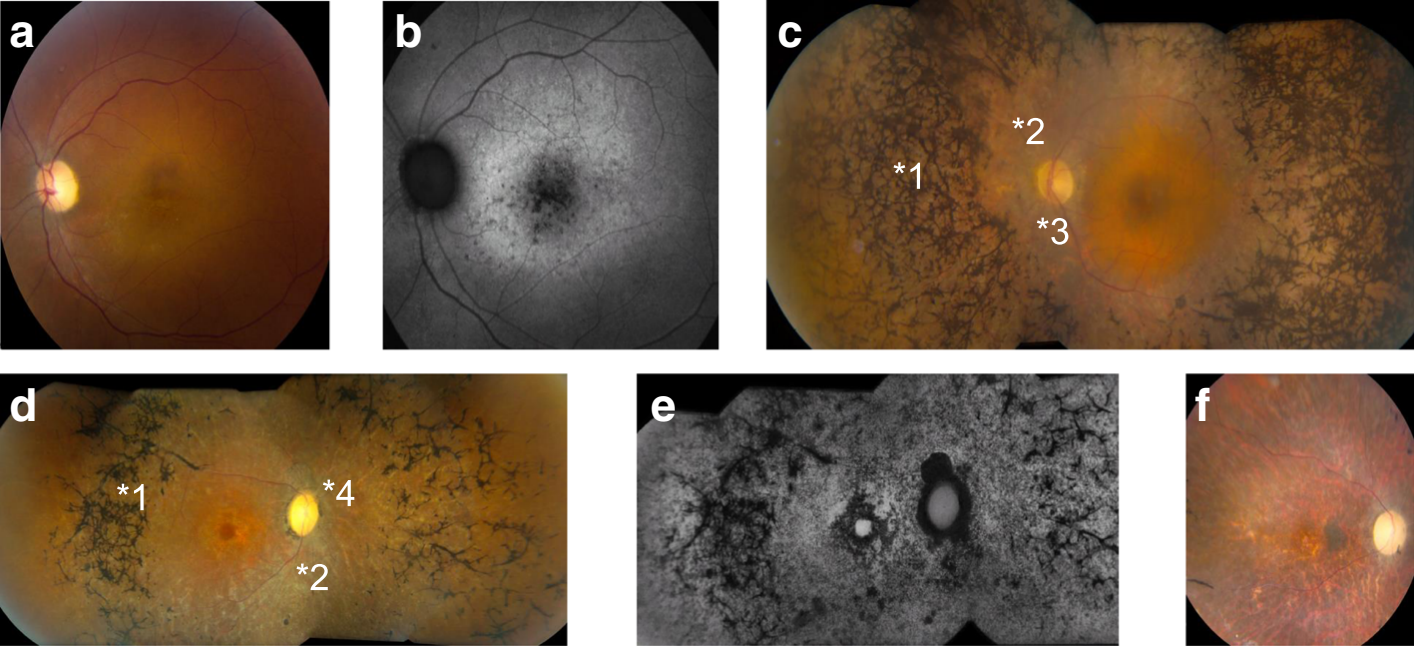
**a** Color and **b** Fundus Autofluorescence (FAF) images of the left eye of a 36 yr. female patient (RD1–12) presenting consistency with a cone-rod dystrophy rather than the reported retinitis pigmentosa that has been described with the ***C2orf71*** gene in the literature. **c** Montage (3-field) color funduscopic image of the left eye of a 55 yr. female patient (RD11–05) with retinitis pigmentosa showing diffuse bony spicules (*1), vascular attenuation (*2), and optic nerve pallor (*3). **d** Color and **e** FAF images of the right eye of a 48 yr. female patient (RD14–05) with advanced rod-cone dystrophy showing macular atrophic changes, severely attenuated vessels (*2), mottling of the retinal pigment epithelium and bony spicules (*1), as well as optic nerve head waxy pallor (*4). **f** Right eye funduscopic appearance of a 74 yr. female patient (RD11–06) with advanced retinal degeneration

We found inconsistences between the genotypes and the clinical diagnoses in 4 samples (Table 2). The confirmed mutations in these 4 patients had been reported with different retinal disorders for the genotype-phenotype relationship in the literature [13–15], but not reported for conditions that our patients were diagnosed with. Patient RD1–12 was diagnosed with sporadic CRD. Two *C2orf71* gene pathogenic variants were confirmed: the p.W505* was reported in unrelated patient [15] and the p.S1090Ifs*17 was novel and not found in populations (ExAC: Exome Aggregation Consortium, Cambridge, MA) (http://exac.broadinstitute.org/). Additionally, three heterozygous stop-gain mutations in unrelated genes (*BBS4, TYRP1*, and *SLC45A2*) were found (Table 2). The *C2orf71* gene mutations were reported in patients with autosomal recessive RP [16], not CRD. Our review of the patient’s clinical conditions still concluded a diagnosis of CRD (Fig. 2a and b). Patient RD11–05 was diagnosed with sporadic RP. Exam was positive for a preserved central visual field island with 20/32 acuity and some macular cystic changes. Electroretinography was consistent with retinitis pigmentosa. We identified a known mutation in the *GUCA1A* gene, p.P50L, which has been reported in patients with autosomal dominant CRD (Downes et al. 2001), not RP. Her clinical manifestation was consistent with RP (Fig. 2c). Patient RD14–05 was diagnosed with sporadic RP. Best corrected visual acuity measured at 20/100 and central visual field was limited to 10 degrees with a few peripheral islands. Findings were consistent with advanced RP (Fig. 2d and e). We found two variants in the *TRPM1* gene, p.W398R and p.R1305H. The p.W398R was novel and was predicted as pathogenic, but the p.R1305 was found in African population with an allele frequency of 2% in our most recent ExAC search (rs13380059). Patient RD11–06 was also diagnosed with RP at age of 74 yr. and no family history was reported. Best corrected visual acuity was at 20/800. The patient reported a 10 year history of decline in peripheral and central visual function and denies any problems with vision as a child. Visual field was limited to a paracentral small island and electroretinography responses were extinguished (Fig. 2f). The patient was found carrying one candidate allele per gene in three genes including 2 reported mutations and 1 reported variant in the dbSNP database in heterozygous state. One of the reported mutations, p.P575L in the *GUCY2D* gene, has a very high allele frequency in the African population in ExAC (unlikely pathogenic). The other known mutation, p.P406L in the *TYR* gene, has been reported in patients with autosomal recessive Oculocutaneous Albinism Type I. The reported variant, IVS9 + 1G > A in the *TYRP1* gene, is predicted as pathogenic for a likely splicing error, but mutations in the *TYRP1* have been reported with autosomal recessive Oculocutaneous Albinism Type III.

**Table 2:** Pathogenic variants with inconsistent clinical correlation

Sample ID	Clincial Diagnosis	Family Inheritance	Candidate Gene(s)	Transcript	Nucleotide Change	Amino Acid Change	Genotypes (Sanger confirmed)	HGMD or dbSNP IDs	References (or in silico Analysis)	
RD1–12	CRD	Sporadic	*C2orf71*	NM_001029883.2	c.1514G > A	p.W505*	Heterozygous	CM1511740		[15]
			*C2orf71*		c.3266dup	p.S1090Ifs*17	Heterozygous	Novel	(predicted as pathogenic	
			*BBS4*	NM_033028.4	c.1375C > T	p.Q459*	Heterozygous	Novel	(predicted as pathogenic)	
			*TYRP1*	NM_000550.2	c.1557 T > G	p.Y519*	Heterozygous	CM135790/rs41302073		[41]
			*GUCA1A*	NM_000409.3	c.149C > T	p.P50L	Heterozygous	CM012969/rs104893968		[13]
RD11–05	RP	Sporadic	*GUCA1A*	NM_000409.3	c.149C > T	p.P50L	Heterozygous	CM012969/rs104893968		[13]
RD14–05	RP	Sporadic	*TRPM1*	NM_002420.5	c.1192 T > C	p.W398R	Heterozygous	Novel	(predicted as pathogenic)	
			*TRPM1*		c.3914G > A	p.R1305H	Heterozygous	rs13380059	*(predicted as unlikely pathogenic*	
RD11–06	RP	Sporadic	*GUCY2D*	NM_000180.3	c.1724C > T	p.P575L	Heterozygous	CM023932/rs28743021		[14]
			*TYR*	NM_000372.4	c.1217C > T	p.P406L	Heterozygous	CM910385/rs104894313		[42]
			*TYRP1*	NM_000550.2	c.1261 + 1G > A	IVS9 + 1G > A	Heterozygous	rs140365820	(predicted as pathogenic)	
*Insufficient for clinical correlation*										
RD4–05	RP	Sporadic	*EYS*	NM_001142800.1	c.6138G > A	p.W2046*	Heterozygous	Novel	(predicted as pathogenic)	
RD11–02	RP	Sporadic	*EYS*	NM_001142800.1	c.5677_5681del	p.Y1893Rfs*12	Heterozygous	Novel	(predicted as pathogenic)	
RD4–03	RP	Sporadic	*FLVCR1*	NM_014053.3	c.1546C > T	p.R516*	Heterozygous	rs538343832	(pathogenic but likely incidental)	
RD14–03	RP	sporadic	*ABCA4*	NM_000350.2	c.5714 + 5G > A	IVS40 + 5G > A	Heterozygous	CS982057		[43]
			*USH2A*	NM_206933.2	c.8600C > T	p.S2867 L	Heterozygous	rs145468090	(predicted as pathogenic)	
			*USH2A*		c.10552G > A	p.V3518I	Heterozygous	rs75397806	*(predicted as unlikely pathogenic)*	
RD15–01	RP	Autosomal recessive	*ABCA4*	NM_000350.2	c.4685 T > C	p.I1562T	Heterozygous	CM970013		[44]
			*RPGRIP1*	NM_020366.3	c.1753C > T	p.P585S	Heterozygous	CM111852		[45]
			*LRP5*	NM_002335.3	c.4574C > T	p.A1525V	Heterozygous	CM078457		[46]
RD6–06	RP	sporadic	*CNGB1*	NM_001297.4	c.2957A > T	p.N986I	Heterozygous	CM111413/rs201162411	(predicted as likely pathogenic)	
			*MYO7A*	NM_000260.3	c.1132C > T	p.R378C	Heterozygous	rs199818783	(predicted as likely pathogenic)	
RD6–02	RP	Sporadic	*RIMS1*	NM_014989.5	c.3027 T > C	p.=	Heterozygous	Novel	(predicted as affecting splicing)	
			*LCA5*	NM_181714.3	c.2050G > C	p.A684P	Heterozygous	rs745875716	*(predicted as unlikely pathogenic)*

We also found single heterozygous mutation status as insufficient to confirm the genotype-phenotype relationship in another 5 patients. The patients RD4–05 and RD11–02 were found heterozygous for novel *EYS* gene mutations, p.W2046* and p.Y1893Rfs*12 respectively. The mutations in the *EYS* gene have been reported with autosomal recessive RP. Since possible gene or partial gene copy number variation or deep intronic mutations were not excluded in this study for these patients, it was insufficient to conclude the correlation in these 2 patients. A similar situation presented itself for patients RD14–03 and RD15–01 who carried single heterozygous mutations in the *ABCA4* gene, IVS + 5G > A splicing error and p.I1562T respectively (Table 2). It was reported that a portion of patients with the Stargardt disease were bearing single heterozygous mutations in the *ABCA4* gene [17, 18]. It could be considered as consistent with the clinical diagnosis of RP, even if the second mutation in their ABCA4 gene was not identified. Adding further complexity to the interpretation, patient RD14–03 was also found carrying two additional *USH2A* gene variants, p.S2867 L and p.V3518I. Of these *USH2A* gene variants, the p.S2867 L has a very low allele frequency in populations (ExAC) (http://exac.broadinstitute.org/) and is predicted as pathogenic, while the p.V3518I has an allele frequency of 2% in the African population and 6 homozygous individual have been identified in the African population(ExAC) (http://exac.broadinstitute.org/). The disease-responsible gene for patient RD14–03 remains unidentified. The patient RD15–01 was found with additional pathogenic variant in the *RPGRIP1* gene, p.P585S - previously reported in patient with autosomal recessive CRD [19].

We have also identified additional previously reported pathogenic variants in some of the probands for whom we established genotype-phenotype relationship in this study (Table 3). These additional pathogenic alleles could be sufficient for a clinical correlation by itself (RD14–08 and RD12–02), or have been reported multiple times in literature (RD20–07, RD12–02, RD12–01, RD13–02, RD12–07, and RD13–08, or are predicted as obligate disease causing variants (RD11–03). For example, the p.C984R in the *GUCY2D* gene was not reported in the literature and predicted as pathogenic. Mutations in the *GUCY2D* have been reported in autosomal dominant CRD. The p.C984R is a valid pathogenic variant by itself.

**Table 3:** Additional pathogenic variants presented in patients may not be relevent

Sample ID	Clincial Diagnosis	Family Inheritance	Candidate Gene(*s)*	Transcript	Nucleotide Change	Amino Acid Change	Genotypes (Sanger confirmed)	HGMD or dbSNP IDs	References (or in silico Analysis)	
RD11–03	RP	Autosomal dominant	*RHO*	NM_000539.3	c.574dup	p.Y192Lfs*139	Heterozygous	Novel	(predicted as pathogenic)	
			*CNGA3*	NM_001298.2	c.1810C > T	p.Q604*	Heterozygous	Novel	(predicted as pathogenic)	
RD20–07	CRD	Autosomal dominant	*PRPH2*	NM_000322.4	c.514C > T	p.R172W	Heterozygous	CM930639		[28]
			*CERKL*	NM_001030311.2	c.847C > T	p.R283*	Heterozygous	CM040509/rs121909398		[47]
RD14–08	RP	Autosomal recessive	*USH2A*	NM_206933.2	c.2276G > T	p.C759F	Heterozygous	CM001372		[30]
			*USH2A*		c.4108G > C	p.V1370 L	Heterozygous	Novel	(predicted as pathogenic)	
			*GUCY2D*	NM_000180.3	c.2950 T > C	p.C984R	Heterozygous	Novel	(predicted as pathogenic)	
RD12–02	XLRP	X-linked	*RPGR*	NM_000328.2	c.1088_1089delinsA	p.V363Dfs*18	Hemizygous	Novel	(predicted as pathogenic)	
			*CACNA1F*	NM_005183.3	c.1619 T > C	p.F540S	Hemizygous	Novel	(predicted as pathogenic)	
			*TMEM67*	NM_153704.5	c.1387C > T	p.R463*	Heterozygous	CM110634		[48]
RD12–01	RP	Sporadic	*RPGR*	NM_000328.2	c.197A > G	p.Q66R	Hemizygous	Novel	(predicted as pathogenic)	
			*TYR*	NM_000372.4	c.721G > A	p.A241T	Heterozygous	CM145799	(DM?)/rs538081629	[49]
RD13–02	RP	Sporadic	*RHO*	NM_000539.3	c.68C > A	p.P23H	Heterozygous	CM900197		[36]
			*ABCA4*	NM_000350.2	c.1610G > A	p.R537H	Heterozygous	CM032805		[50]
RD12–07	RP	Sporadic	*RHO*	NM_000539.3	c.936 + 1G > T	IVS4 + 1G > T	Heterozygous	CS920776		[37]
			*TYRP1*	NM_000550.2	c.1354A > G	p.M452 V	Heterozygous	CM081465		[51]
RD13–08	Achromatopsia	Sporadic	*CNGB3*	NM_019098.4	c.1148delC	p.T383Ifs*13	Heterozygous	CD001927		[52]
			*ABCA4*	NM_000350.2	c.6089G > A	p.R2030Q	Heterozygous	CM990070/rs61750641		[53]
			*RPGRIP1*	NM_020366.3	c.1767G > T	p.Q589H	Heterozygous	CM057749/rs34067949		[45]

To technically verify the sequencing results, we included 4 previously tested DNA samples. Two of them were tested in the resequencing chip by Affimatrix, in which we had previously identified and validated one missense mutation in the *GUCY2D* gene (RD1–01) and no mutation in a Coriell DNA sample RD1–03, ND0068*B1 [12]. The other 2 samples were from previous Sanger sequencing: one 1 bp deletion in the *CNGB3* gene in one sample (RD13–08) and two OCA gene mutations in the other sample (RD13–07). All of the mutations were correctly identified through the bioinformatics pipeline. In addition to the previously identified mutation, we also detected additional pathogenic variants in the RD13–08 (Table 3).

## Discussion

Microdroplet PCR has been used in many studies including the sequencing of the entire human X chromosome exome with coverage of 97% [11]. Our results demonstrated as much as 99% coverage and average read depth at 119X in our standardized procedure. We were testing the microdroplet PCR as the enrichment method for the targeted gene panel NGS with consideration of repeatability, standardization, and potential clinical application. Comparing with other enrichment technologies at the time, the microdroplet PCR provided a reliable and manageable procedure. As we observed random low coverage of a number of target exons with the capture based enrichment technologies in our studies and published data, we could address the uncovered target exons with a simultaneously pooled supplementary PCR using individually designed alternative primer pairs and unique PCR conditions. In addition, the designed primer library could be quickly adapted to verify the variants found in NGS by Sanger sequencing or non-amplifiable targets in target samples. The disadvantage of the microdroplet PCR included high up-front costs for the equipment and annual maintenance service contract even if there was no high volume sample flow.

After comparing several public and commercial data processing softwares, we chose the Qiagen Genomics Workbench as our primary sequence aligner and annotation tool. For quality control purposes, beside the general alignment and coverage analysis, this software provided a detailed amplicon based analysis table including reads per target region (amplicon), percent sequence coverage per amplicon, and depth of reads as zero covered target bases, reads at minimally covered bases, reads at maximally covered and averages of reads at non-zero covered sequences, which easily determined the amplification resistant targets. It enabled a standardized protocol with clear information on exact coverage limitations for every sample. As a microdroplet PCR based NGS, we obtained a comprehensive list of targets with no amplification. It will enable 100% coverage with follow-up Sanger sequencing (or other approaches) on targets with poor coverage quality if necessary. Although we could not extend the analysis to the copy number variation with large deletions/insertions beyond the size of amplicons in this study, we are still looking for the potential solution by implementing other aligner and calculating Algorithms.

Many NGS studies have been published recently to report pathogenic variant found in IRD patients [6–10]. NGS has the power to screen hundreds of genes in one reaction thereby increasing of finding incidental pathogenic variants. It will be essential to analyze the clinical validity by clinical correlation, family mutation co-segregation analysis, or other approaches to determine if the pathogenic alleles were indeed the disease-causing mutations per patient. Reporting pathogenic variants does not necessarily provide direct answers to the question regarding patient specific disease-causing mutations. Making studies available in literature could help other clinical geneticists by providing real cases with consistent clinical correlations and more importantly, detailed discussions of inconsistent/insufficient correlations.

**Fig. 3:**
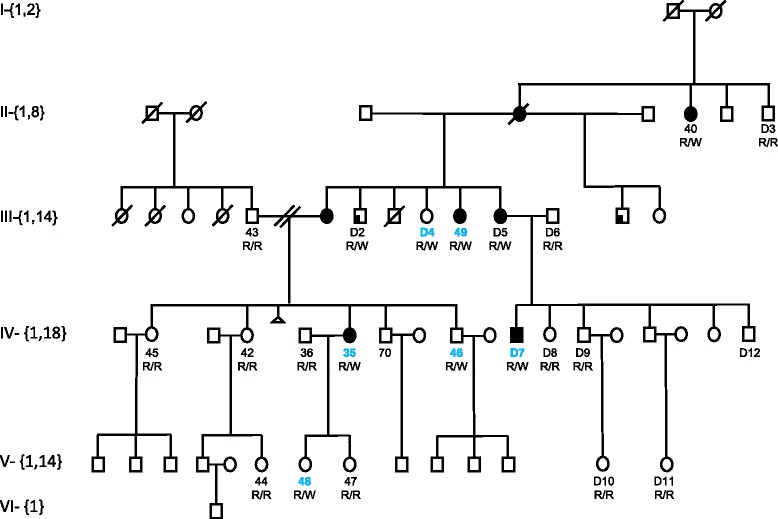
OCMD in a family with incomplete penetrance. Each individual family member was identified with an assigned number ID (such as 40, 43, 45, 42, 36, 35, 70, 46, 44, 48, and 47) or a number after D (such as D2-D12). The genotypes of the *RP1L1* gene, p.R45W mutation has been determined in every member if their DNA was available and labeled as R/R for homozygous of R45 allele or R/W for heterozygous of the p.R45W mutation. The samples with colored IDs have been analyzed in this NGS study. The corresponding IDs were: D4 = RD10–01; D7 = RD10–02; 35 = RD10–03; 46 = RD10–04; 48 = RD10–05; 49 = RD10–06, in the NGS analysis. For symbols,  represents additional condition not related to retinal dystrophy;  represents miscarriage

In this study, we had the chance to evaluate pathogenic variants in the context of clinical presentations in these patients. We determined that 38 out of 67 probands had consistent clinical correlations (56.7%, Table 1). Segregation analysis and testing unaffected family members was performed wherever possible. Patients RD13–01 and RD14–04 were CRD affected son and affected mom. They were both heterozygous for the *GUCA1A* p.Y99C. Patients RD2–01 and RD2–02 are affected mom and daughter with peripapillary atrophy. There are two additional affected males in this family indicating a dominant inheritance (Leys et al., personal communication, manuscript is in preparation separately). It was interesting to note that patients RD10–02, RD10–03, and RD10–06 were three affected OCMD belonging to a single large family with clear autosomal dominant inheritance (Fig. 3). Six family members were included in this study. The other three family members (RD10–01, RD10–04, and RD10–05) were determined as *RP1L1* p.R45W pathogenic variant positive and no clinical presentation of OCMD at the time was examined in the past. We included them to test the potential contribution of other pathogenic variants in the retinal genes. No additional pathogenic variant was found in the three affected patients in this family and no apparent co-segregation of any variants within two groups (affected three vs unaffected three). This result is consistent with the previously reported findings of incomplete penetrance of the p.R45W mutation in OCMD [20, 21]. The potential genetic modification for an in-family variation was unlikely from other retinal function related genes included in this study (Additional file 1: Table S1). We suspected that the p.R45W mutation might rather be a gain-of-function from malfunctioned products by an interaction with unknown component, which could lead to late onset macular dystrophy. The modification could be from a restricted expression of the wild type products too.

We found *USH2A* p.C759F mutation in a couple of RP families. Two patients, RD20–08 and RD20–02 were from a family with confirmed compound heterozygosity of the most common *USH2A* gene mutation, p.E766Sfs*21 and the p.C759F. Carrier status of the p.C759F was also confirmed in one available parent (RD20–01) in this family. In the other family, patient RD5–07 was apparently homozygous of the p.C759F. The *USH2A* gene mutations have been reported in the Usher syndrome and non-syndromic RP [22, 23]. The p.C759F was presumed as retinal disease specific allele [23]. It was consistent in this study that the p.C759F correlated with Nonsyndromic RP. However, the pathogenicity of homozygous p.C759F was challenged by a recent clinical report that an unaffected and one affected dizygotic twin brothers shares homozygosity of p.C759F [24].

Many studies report putative pathogenic variants in genes that lack established correlation with the patients’ clinical manifestations [6, 15]. The inconsistencies surely raise the question each time about the correlation and deserve a careful review. Making these analyses available to our community would be of great interest for clinical diagnostic practice because of the impact on the interpretation for the clinical diagnostic laboratories. With the capacity of NGS, we will encounter cases with similar scenarios from time to time. In this study, the pathogenic variants overrepresented in patient RD1–12 could not be explained with the clinical manifestations of CRD (Table 2 and Fig. 2a and b). Two *EYS* gene pathogenic variants would be interpreted as putative disease causing in patients with RP as a clinical diagnosis, but those do not correlate with the CRD diagnosis in the patient RD1–12. On the other hand, we don’t know whether the additional pathogenic variants could contribute to the etiology or pathology of the clinical presentation in this patient. The patient RD11–05 was diagnosed with RP (Fig. 2 and Table 2) and we only found the *GUCA1A* p.P50L, which was reported in a family with autosomal dominant CRD and marked variability in expressivity [13], not RP. The ExAC reported an allele frequency of 0.1%, which was too high for an autosomal dominant inheritance. It could be possible that the p.P50L was coincidentally co-segregating with the CRD in the published CRD family if we disqualified the pathogenicity. In our patient RD11–05, it again could coincidentally co-segregate with RP. We should pay attention to the correlation if more cases with the same segregation can be found. The mutations in the *TRPM1* gene have been reported in autosomal recessive Congenital Stationary Night Blindness [25]. The patient RD14–05 was reviewed for clinical presentations and we could not correlate the two *TRPM1* gene variants to her clinical manifestations (Table 3). The inconclusive clinical correlation did not support the predicted pathogenicity. For the patient RD11–06, the high frequent *GUCY2D* gene p.P575L allele does not support the pathogenicity. Two mutations in two different OCA related genes have no direct clinical relevance to the RP diagnosis in this patient. In our designing of this gene panel, we included 7 genes related to OCA. It was interesting to point out that we had observed relative high frequencies of OCA related alleles in heterozygous in 10 samples of this cohort (total 12 alleles were all in patient samples, not in the controls or family members except the OCA control sample) (Additional file 4: Table S4). We were not sure if this was just by chance or there was indeed an overrepresentation of OCA related alleles in IRD patients with certain phenotypes.

In our review of the clinical diagnosis of the 11 patients with inconclusive pathogenic variants in Table 2, we noticed comparable genotypes in some of the patients listed in Table 1 with the additional pathogenic variants. The combined genotypes were summarized in Table 3. If we made an assumption that the patients in Table 2 could have disease responsible genes not included in this study, those pathogenic variants we found in Table 2 might represent as the additional pathogenic variants like those in Table 3. It would be difficult to distinguish the two possibilities. We would assume that the likelihood in a patient with additional pathogenic variant being found and the disease-causing mutation being not found is much less likely based on overall positive clinical correlations in all studies. Interestingly, we found that one of the control samples also carried additional pathogenic variants in *ABCA4* gene and *RPGRIP1* gene and this sample was from a patient who was diagnosed with Achromatopsia (Table 3, RD13–08).

In addition, it is too early to suggest any pathogenic contribution from these additional pathogenic variants. In the 18 probands that had no potential pathogenic variant identified in this study, one patient was clinically diagnosed with Gyrate Atrophy and another one with Leber’s Hereditary Optic Neuropathy in our follow-up clinical review.

## Conclusions

In summary, we tested a 184 targeted gene panel sequencing in 74 patients with different inheritance patterns and clinical diagnoses. We demonstrated that next-generation sequencing can be an effective tool for determining the pathogenic variants in inherited disease families with highly heterogeneous causes. We highlight the importance of interpreting molecular findings in the context of patient clinical presentation.

## Additional files

Additional file 1: Table S1. Summary of genes and their clinical correlation. A panel of 184 RD genes responsible for retinal related diseases was provided. (PDF 45 kb)

Additional file 2: Table S2. Summary of Retinal Dystrophy primer library design. Statisitc analysis of designing for the primer sets covering entire coding and intronic flanking regions. (PDF 8 kb)

Additional file 3: Table S3. Summary of candidate variants in this cohort. A complete list of candidate variants that found in this patient group before clinical correlation was established. (PDF 301 kb)

Additional file 4: Table S4. Pathogenic variants in OCA related genes. Carrying status of pathogenic variants in the OCA genes in some of the RDS patients. (PDF 275 kb)

## Abbreviations

AD: Autosomal dominant
AR: Autosomal recessive
CRD: Cone-rod dystrophy
RD: Retinal dystrophy
RP: Retinitis pigmentosa
SNP: Single nucleotide polymorphism
STGD: Stargardt disease
XL: X-linked
