# Supplementary material for: Applying next generation sequencing with microdroplet PCR to determine the disease-causing mutations in retinal dystrophies
Source: BMC Ophthalmol. 2017 Aug 24;17:157. doi: 10.1186/s12886-017-0549-5 (PMC5571584; doi:10.1186/s12886-017-0549-5)
Supplement: Supplementary file 1 — Summary of genes and their clinical correlation. A panel of 184 RD genes responsible for retinal related diseases was provided. (PDF 45 kb) [file 12886_2017_549_MOESM1_ESM.pdf]

**Supplementary Table 1. Summary of genes and their clinical correlation**

| Clinical classes of retinal disorders | Disease inheritable genes* | | | | | | | | | | | | | | | |
|---|---|---|---|---|---|---|---|---|---|---|---|---|---|---|---|---|
| Bardet-Biedl syndrome, autosomal recessive | *ARL6* | *BBS1* | *BBS2* | *BBS4* | *BBS5* | *BBS7* | *BBS9* | *BBS10* | *BBS12* | *CEP290* | *INPP5E* | *MKKS* | *MKS1* | *TRIM32* | *TTC8* | |
| Chorioretinal atrophy or degeneration autosomal dominant | *RGR* | *TEAD1* | | | | | | | | | | | | | | |
| Cone or cone-rod dystrophy, autosomal dominant | *AIPL1* | *CRX* | *GUCA1A* | *GUCY2D* | *PITPNM3* | *PROM1* | *PRPH2* | *RIMS1* | *SEMA4A* | *UNC119* | | | | | | |
| Cone or cone-rod dystrophy, autosomal recessive | *ABCA4* | *ADAM9* | *CACNA2D4* | *CDHR1* | *CERKL* | *CNGB3* | *KCNV2* | *PDE6C* | *RAX2* | *RDH5* | *RPGRIP1* | | | | | |
| Cone or cone-rod dystrophy, X-linked | *CACNA1F* | *RPGR* | | | | | | | | | | | | | | |
| Congenital stationary night blindness, autosomal dominant | *GNAT1* | *PDE6B* | *RHO* | | | | | | | | | | | | | |
| Congenital stationary night blindness, autosomal recessive | *CABP4* | *GRK1* | *GRM6* | *RDH5* | *SAG* | *SLC24A1* | *TRPM1* | | | | | | | | | |
| Congenital stationary night blindness, X-linked | *CACNA1F* | *NYX* | | | | | | | | | | | | | | |
| Deafness alone or syndromic, autosomal dominant | *WFS1* | | | | | | | | | | | | | | | |
| Deafness alone or syndromic, autosomal recessive | *CDH23* | *DFNB31* | *MYO7A* | *PCDH15* | *PDZD7* | *USH1C* | | | | | | | | | | |
| Leber congenital amaurosis, autosomal dominant | *CRX* | *IMPDH1* | *OTX2* | | | | | | | | | | | | | |
| Leber congenital amaurosis, autosomal recessive | *AIPL1* | *CABP4* | *CEP290* | *CRB1* | *CRX* | *GUCY2D* | *IQCB1* | *LCA5* | *LRAT* | *RD3* | *RDH12* | *RPE65* | *RPGRIP1* | *SPATA7* | *TULP1* | |
| Macular degeneration, autosomal dominant | *BEST1* | *C1QTNF5* | *EFEMP1* | *ELOVL4* | *FSCN2* | *GUCA1B* | *HMCN1* | *PROM1* | *PRPH2* | *RP1L1* | *TIMP3* | | | | | |
| Macular degeneration, autosomal recessive | *ABCA4* | *CFH* | | | | | | | | | | | | | | |
| Macular degeneration, X-linked | *RPGR* | | | | | | | | | | | | | | | |
| Ocular-retinal developmental disease, autosomal dominant | *VCAN* | | | | | | | | | | | | | | | |
| Optic atrophy, autosomal dominant | *OPA1* | | | | | | | | | | | | | | | |
| Optic atrophy, autosomal recessive | *TMEM126A* | | | | | | | | | | | | | | | |
| Optic atrophy, X-linked | *TIMM8A* | | | | | | | | | | | | | | | |
| Retinitis pigmentosa, autosomal dominant | *BEST1* | *CA4* | *CRX* | *FSCN2* | *GUCA1B* | *IMPDH1* | *KLHL7* | *NR2E3* | *NRL* | *PRPF3* | *PRPF8* | *PRPF31* | *PRPH2* | *RDH12* | *RHO* | |
| | *ROM1* | *RP1* | *RP9* | *SEMA4A* | *SNRNP200* | *TOPORS* | | | | | | | | | | |
| Retinitis pigmentosa, autosomal recessive | *ABCA4* | *BEST1* | *C2ORF71* | *CERKL* | *CLRN1* | *CNGA1* | *CNGB1* | *CRB1* | *DHDDS* | *EYS* | *FAM161A* | *IDH3B* | *IMPG2* | *LRAT* | *MERTK* | |
| | *NR2E3* | *NRL* | *PDE6A* | *PDE6B* | *PDE6G* | *PRCD* | *PROM1* | *RBP3* | *RGR* | *RHO* | *RLBP1* | *RP1* | *RPE65* | *SAG* | *SPATA7* | |
| | *TTC8* | *TULP1* | *USH2A* | *ZNF513* | | | | | | | | | | | | |
| Retinitis pigmentosa, X-linked | *RP2* | *RPGR* | | | | | | | | | | | | | | |
| Syndromic/systemic diseases with retinopathy, autosomal dominant | *ABCC6* | *ATXN7* | *COL11A1* | *COL2A1* | *JAG1* | *KCNJ13* | *PAX2* | *TREX1* | *VCAN* | | | | | | | |
| Syndromic/systemic diseases with retinopathy, autosomal recessive | *ABCC6* | *AHI1* | *ALMS1* | *CC2D2A* | *CEP290* | *CLN3* | *COL9A1* | *FLVCR1* | *INPP5E* | *INVS* | *IQCB1* | *LRP5* | *MKS1* | *MTTP* | *NPHP1* | |
| | *NPHP3* | *NPHP4* | *OPA3* | *PANK2* | *PEX1* | *PEX7* | *PHYH* | *PXMP3* | *RPGRIP1L* | *SDCCAG8* | *TTPA* | *WFS1* | | | | |
| Syndromic/systemic diseases with retinopathy, X-linked | *OFD1* | *TIMM8A* | | | | | | | | | | | | | | |
| Usher syndrome, autosomal recessive | *CDH23* | *CLRN1* | *DFNB31* | *GPR98* | *MYO7A* | *PCDH15* | *USH1C* | *USH1G* | *USH2A* | | | | | | | |
| Other retinopathy, autosomal dominant | *BEST1* | *CRB1* | *FZD4* | *LRP5* | *OPN1SW* | *RB1* | *TSPAN12* | | | | | | | | | |
| Other retinopathy, autosomal recessive | *BEST1* | *CDH3* | *CNGA3* | *CNGB3* | *CNNM4* | *CYP4V2* | *GNAT2* | *LRP5* | *MFRP* | *NR2E3* | *OAT* | *PROM1* | *RBP4* | *RGS9* | *RGS9BP* | RLBP1 |
| Other retinopathy, X-linked | *CACNA1F* | *CHM* | *DMD* | *NDP* | *OPN1LW* | *OPN1MW* | *PGK1* | *RS1* | | | | | | | | |
| Eye development related genes | *PAX6* | *CHD7* | *TMEM67* | *MKS1* | *HMX1* | *GDF6* | *GDF5* | *BMP4* | *BMP7* | *VSX2* | *MITF* | *NLZ1* | *NLZ2* | | | |
| Pigmentation genes | *TYR* | *OCA2* | *TYRP1* | *SLC45A2* | *SLC24A4* | *ASIP* | *MC1R* | | | | | | | | | |

Note: *Some of the genes appeared in two or more classes of disorders.
