# Supplementary material for: Applying next generation sequencing with microdroplet PCR to determine the disease-causing mutations in retinal dystrophies
Source: BMC Ophthalmol. 2017 Aug 24;17:157. doi: 10.1186/s12886-017-0549-5 (PMC5571584; doi:10.1186/s12886-017-0549-5)
Supplement: Supplementary file 2 — Summary of Retinal Dystrophy primer library design. Statisitc analysis of designing for the primer sets covering entire coding and intronic flanking regions. (PDF 8 kb) [file 12886_2017_549_MOESM2_ESM.pdf]

Supplementary Table 2. Summary of Retinal Dystrophy primer library design

Candidate genes for targeted sequencing	184
Number of submitted targets	2,827
Number of exons in complete design	2,078
Number of amplicons in complete design	3071
Total number of amplicon bases in complete design	921,989
Total number of unique loci bases in complete design	560,253
Percent of unique loci bases covered	99.89%
Number of MP* amplicons in complete design	35

Note: *Primers can be mapped to multiple locations in the genome due to sequence similarity. Primers of genes which can be mapped to multiple locations are as follows: OPN1LW, OPN1MW.
