# Supplementary material for: Applying next generation sequencing with microdroplet PCR to determine the disease-causing mutations in retinal dystrophies
Source: BMC Ophthalmol. 2017 Aug 24;17:157. doi: 10.1186/s12886-017-0549-5 (PMC5571584; doi:10.1186/s12886-017-0549-5)
Supplement: Supplementary file 3 — Summary of candidate variants in this cohort. A complete list of candidate variants that found in this patient group before clinical correlation was established. (PDF 301 kb) [file 12886_2017_549_MOESM3_ESM.pdf]

**Supplementary Table 3. Summary of candidate variants in this cohort**

| Sample ID | Candidate Gene(s) | Transcript | Nucleotide Change | Amino Acid Change | Genotypes (Sanger confirmed) | HGMD or dbSNP IDs | References |
|---|---|---|---|---|---|---|---|
| RD13-01 | *GUCA1A* | NM_000409.3 | c.296A>G | p.Y99C | Heterozygous | CM980960 | Payne et al. [1998] |
| RD1-07 | *PRPF31* | NM_015629.3 | c.220C>T | p.Q74* | Heterozygous | CM063073 | Sullivan et al. [2006] |
| RD20-06 | *PRPH2* | NM_000322.4 | c.514C>T | p.R172W | Heterozygous | CM930639 | Poloschek et al. [2010] |
| RD20-07 | *PRPH2* | NM_000322.4 | c.514C>T | p.R172W | Heterozygous | CM930639 | Poloschek et al. [2010] |
| RD2-01 | *RGR* | NM_001012720.1 | c.824dupG | p.I276N*77 | Heterozygous | CI993291 | Morimura et al. 1999 |
| RD11-03 | *RHO* | NM_000539.3 | c.574dup | p.Y192Lfs*139 | Heterozygous | Novel | |
| RD14-07 | *TOPORS* | NM_005802.4 | c.2474dupA | p.Y825* | Heterozygous | CSI075704 | Chakarova et al [2007] |
| RD10-02 | *RP1L1* | NM_178857.5 | c.133C>T | p.R45W | Heterozygous | CM105618 | Akahori et al [2010] |
| RD5-08 | *CERKL* | NM_001030311.2 | c.1045_1046del | p.M349Vfs*20 | Heterozygous | Novel | |
| | | | c.316C>T | p.R106C | Heterozygous | Novel | |
| RD14-08 | *USH2A* | NM_206933.2 | c.2276G>T | p.C759F | Heterozygous | CM001372 | Garcia-Garcia et al. [2011] |
| | | | c.4108G>C | p.Va370L | Heterozygous | Novel | |
| RD20-08 | *USH2A* | NM_206933.2 | c.2299delG | p.E766Sfs*21 | Heterozygous | CD982997 | O'Sullivan et al. [2012] |
| | | | c.2276G>T | p.C759F | Heterozygous | CM001372 | Garcia-Garcia et al. [2011] |
| RD20-05 | *CACNA1F* | NM_005183.3 | c.2576+1G>A | IVS20+1G>A | Hemizygous | Novel | |
| RD6-08 | *CHM* | NM_000390.2 | c.49+2dupT | IVS1+2dupT | Hemizygous | CI137323 | Ramsden et al. [2013] |
| RD12-02 | *RPGR* | NM_000328.2 | c.1088_1089delinsA | p.V363Dfs*18 | Hemizygous | Novel | |
| RD4-04 | *ABCA4* | NM_000350.2 | c.1804C>T | p.R602W | Homozygous | CM990025 | Riveiro-Alvarez et al. [2013] |
| RD6-01 | *BBS1* | NM_024649.4 | c.1169T>G | p.M390R | Homozygous | CM021489 | Bealse et al. [2003] |
| RD12-06 | *CERKL* | NM_001030311.2 | c.481+2T>G | IVS2+2T>G | Homozygous | CS140556 | Glockle et al. [2013] |
| RD4-06 | *EYS* | NM_001142800.1 | c.2259+1G>A | IVS14+1G>A | Heterozygous | CS150721 | Bonilha et al. [2014] |
| | | | c.6137G>A | p.W2046* | Heterozygous | Novel | |
| RD6-07 | *EYS* | NM_001142800.1 | c.8473_8474insT | p.V2804fs | Heterozygous | Novel | |
| | | | c.1153T>G | p.C385G | Heterozygous | Novel | |
| RD12-05 | *EYS* | NM_001142800.1 | c.6416G>A | p.C2139Y | Heterozygous | CM102730 | Audo et al. [2010] |
| | | | c.7868G>A | p.G2623E | Heterozygous | Novel | |
| RD13-05 | *GUCY2D* | NM_000180.3 | c.2375C>T | p.P792L | Heterozygous | rs763774686 | |
| RD11-08 | *IMPDH1* | NM_000883.3 | c.931G>A | p.D311N | Heterozygous | CM020283 | Bowne et al. [2002] |
| RD15-03 | *IMPDH1* | NM_000883.3 | c.931G>A | p.D311N | Heterozygous | CM020283 | Bowne et al. [2002] |
| RD5-04 | *RHO* | NM_000539.3 | c.68C>A | p.P23H | Heterozygous | CM900197 | Dryja et al [1990] |
| RD13-02 | *RHO* | NM_000539.3 | c.68C>A | p.P23H | Heterozygous | CM900197 | Dryja et al [1990] |
| RD12-08 | *RHO* | NM_000539.3 | c.561T>G | p.C187W | Heterozygous | Novel | |
| RD12-07 | *RHO* | NM_000539.3 | c.936+1G>T | IVS4+1G>T | Heterozygous | CS920776 | Hernan et al. [2011] |
| RD14-06 | *PRPH2* | NM_000322.4 | c.514C>T | p.R172W | Heterozygous | CM930639 | Poloschek et al. [2010] |
| RD12-03 | *RPE65* | NM_000329.2 | c.886dup | p.R296Kfs*7 | Homozygous | CI107001 | Coppieters et al. [2010] |
| RD12-01 | *RPGR* | NM_000328.2 | c.197A>G | p.Q66R | Hemizygous | Novel | |
| RD6-05 | *TIMP3* | NM_000362.4 | c.29T>A | p.L10H | Heterozygous | Novel | |
| RD20-03 | *TRPM1* | NM_002420.5 | c.1197G>A | p.P421= | Heterozygous | CS097758 | Audo et al. [2009] |
| | | | c.215A>G | p.Y72C | Heterozygous | CM097760 | Audo et al. [2009] |
| RD6-04 | *TRPM1* | NM_002420.5 | c.2947_2948delGCinsAT | p.A983I | Heterozygous | Novel | |
| | | | c.3125T>G | p.L1042R | Heterozygous | Novel | |
| RD14-02 | *USH2A* | NM_206933.2 | c.11411del | p.P3804Lfs*13 | Heterozygous | CD149996 | Baux et al. [2014] |
| | | | c.8431C>A | p.P2811T | Heterozygous | rs111033529 | |
| RD11-04 | *USH2A* | NM_206933.2 | c.13335_13347delinsCTTG | p.E4445_S4449delinsDL | Heterozygous | CX104126 | McGee et al. [2010] |
| | | | c.14402_14403del | p.Y4801Ffs*20 | Heterozygous | Novel | |
| RD14-04 | *GUCA1A* | NM_000409.3 | c.296A>G | p.Y99C | Heterozygous | CM980960 | Payne et al. [1998] |
| RD2-02 | *RGR* | NM_001012720.1 | c.824dupG | p.I276N*77 | Heterozygous | CI993291 | Morimura et al. [2000] |
| RD10-02 | *RP1L1* | NM_178857.5 | c.133C>T | p.R45W | Heterozygous | CM105618 | Akahori et al [2010] |
| RD10-02 | *RP1L1* | NM_178857.5 | c.133C>T | p.R45W | Heterozygous | CM105618 | Akahori et al [2010] |
| RD18-02 | *CERKL* | NM_001030311.2 | c.1045_1046del | p.M349Vfs*20 | Heterozygous | Novel | |
| | *CERKL* | | c.316C>T | p.R106C | Heterozygous | Novel | |
| RD20-02 | *USH2A* | NM_206933.2 | c.2299delG | p.E766Sfs*21 | Heterozygous | CD982997 | O'Sullivan et al. [2012] |
| | *USH2A* | | c.2276G>T | p.C759F | Heterozygous | CM001372 | Garcia-Garcia et al. [2011] |
| RD1-12 | *C2orf71* | NM_001029883.2 | c.1514G>A | p.W505* | Heterozygous | CM1511740 | Yang et al [2015] |
| | *C2orf71* | | c.3266dup | p.S1090Ifs*17 | Heterozygous | Novel | |
| | *BBS4* | NM_033028.4 | c.1375C>T | p.Q459* | Heterozygous | Novel | |
| | *TYRP1* | NM_000550.2 | c.1557T>G | p.Y519* | Heterozygous | CM135790 | Simeonov et al. [2013] |
| | *SLC45A2* | NM_016180 | c.834C>G | p.Y278* | Heterozygous | CM083852 | Hutton et al. [2008] |
| RD11-05 | *GUCA1A* | NM_000409.3 | c.149C>T | p.P50L | Heterozygous | CM012969 | Downes et al. [2001] |
| RD11-06 | *GUCY2D* | NM_000180.3 | c.1724C>T | p.P575L | Heterozygous | CM023932/rs28743021 | Small et al. [2008] |
| | *TYR* | NM_000372.4 | c.1217C>T | p.P406L | Heterozygous | CM910385/rs104894313 | Giebel et al. [1991] |
| | *TYRP1* | NM_000550.2 | c.1261+1G>A | IVS9+1G>A | Heterozygous | rs140365820 | |
| RD14-05 | *TRPM1* | NM_002420.5 | c.1192T>C | p.W398R | Heterozygous | Novel | |
| | *TRPM1* | | c.3914G>A | p.R1305H | Heterozygous | Novel | |
| RD14-03 | *ABCA4* | NM_000350.2 | c.5714+5G>A | IVS40+5G>A | Heterozygous | CS982057 | Cremers et al. [1998] |
| | *USH2A* | NM_206933.2 | c.8600C>T | p.S2867L | Heterozygous | rs145468090 | |
| | *USH2A* | | c.10552G>A | p.V3518I | Heterozygous | rs75397806 | |
| RD4-05 | *EYS* | NM_001142800.1 | c.6138G>A | p.W2046* | Heterozygous | Novel | |
| RD11-02 | *EYS* | NM_001142800.1 | c.5677_5681del | p.Y1893Rfs*12 | Heterozygous | Novel | |
| RD15-01 | *ABCA4* | NM_000350.2 | c.4685T>C | p.I1562T | Heterozygous | CM970013 | Allikmets et al. [1997] |
| | *RPGRIP1* | NM_020366.3 | c.1753C>T | p.P585S | Heterozygous | CM111852 | Fernandez-Martinez et al. [2011] |
| | *LRP5* | NM_002335.3 | c.4574C>T | p.A1525V | Heterozygous | CM078457 | Guo & Cooper [2007] |
| RD14-01 | *BBS9* | NM_198428.2 | c.1280C>T | p.A427V | Heterozygous | rs138072724 | |
| | *USH1G* | NM_173477.4 | c.1120T>C | p.W374R | Heterozygous | Novel | |
| | *GPR98* | NM_032119.3 | c.5295C>G | p.F1765L | Heterozygous | rs201388114 | |
| RD1-07 | *ABCA4* | NM_000350.2 | c.635G>A | p.R212H | Heterozygous | CM020912 | Pang et al. [2002] |
| | *ABCA4* | | c.6529G>A | p.D2177N | Heterozygous | CM970023 | Wiszniewski et al. [2005] |
| | *ABCA4* | | c.1552G>A | p.E518K | Heterozygous | CM1410571(DM?)/rs369 | Leslie et al. [2015] |
| RD11-03 | *CNGA3* | NM_001298.2 | c.1810C>T | p.Q604* | Heterozygous | Novel | |
| | *RPE65* | NM_000329.2 | c.963T>G | p.N321K | Heterozygous | CM003833/rs149916178 | Philp et al. [2009] |
| RD20-07 | *CERKL* | NM_001030311.2 | c.769C>T | p.R257* | Heterozygous | CM040509 | Bornancin et al. [2005] |
| RD14-08 | *GUCY2D* | NM_000180.3 | c.2950T>C | p.C984R | Heterozygous | Novel | |
| RD20-02 | *CLRN1* | NM_174878.2 | c.218A>G | p.Q73R | Heterozygous | CM127999 | Licastro et al. [2012] |
| RD12-02 | *CACNA1F* | NM_005183.3 | c.1619T>C | p.F540S | Hemizygous | Novel | |
| | *TMEM67* | NM_153704.5 | c.1387C>T | p.R463* | Heterozygous | CM110634 | Chaki et al. [2011] |
| RD12-01 | *RP1L1* | NM_178857.5 | c.416dup | p.G140Rfs*10 | Heterozygous | rs201192645 | |
| | *TYR* | NM_000372.4 | c.721G>A | p.A241T | Heterozygous | CM145799 (DM?) | Fossbakk et al. [2014] |
| RD13-02 | *ABCA4* | NM_000350.2 | c.1610G>A | p.R537H | Heterozygous | CM032805 | Jaakson et al [2003] |

| | | | | | | | |
|---|---|---|---|---|---|---|---|
| **RD12-07** | ***TYRP1*** | NM_000550.2 | c.1354A>G | p.M452V | Heterozygous | CM081465 | Hutton et al. [2008] |
| **RD13-08** | ***ABCA4*** | NM_000350.2 | c.6089G>A | p.R2030Q | Heterozygous | CM990070 | Lewis et al. [1999] |
| | ***RPGRIP1*** | NM_020366.3 | c.1767G>T | p.Q589H | Heterozygous | CM057749 | Fernández-Martínez et al. [2011] |
| | | | | | | | |
| **RD15-03** | ***OCA2*** | NM_000275.2 | c.1004C>T | p.T335M | Heterozygous | rs533988694 | |
| **RD20-03** | ***OCA2*** | NM_000275.2 | c.1513A>C | p.F505V | Heterozygous | None | UCSC:15-28211959-A-C |
| **RD6-05** | ***TYR_1*** | NM_000372.4 | c.740G>T | p.C247F | Heterozygous | Novel | |
| **RD11-07** | ***OCA2*** | NM_000275.2 | c.1574C>T | p.P525L | Heterozygous | None | UCSC:15-2821898-G-A |
| **RD11-08** | ***TYRP1*** | NM_000550.2 | c.977G>A | p.R326H | Heterozygous | CM135784/rs16929374 | |
| **RD13-01** | ***OCA2*** | NM_000275.2 | c.40G>A | p.A14T | Heterozygous | rs368928996 | |
