# Supplementary material for: Applying next generation sequencing with microdroplet PCR to determine the disease-causing mutations in retinal dystrophies
Source: BMC Ophthalmol. 2017 Aug 24;17:157. doi: 10.1186/s12886-017-0549-5 (PMC5571584; doi:10.1186/s12886-017-0549-5)
Supplement: Supplementary file 4 — Pathogenic variants in OCA related genes. Carrying status of pathogenic variants in the OCA genes in some of the RDS patients. (PDF 275 kb) [file 12886_2017_549_MOESM4_ESM.pdf]

Supplementary Table 4. Pathogenic variants in OCA related genes

Sample ID	Family Inheritance	Clincial Diagnosis	Candidate Gene(s)	Transcript	Nucleotide Change	Amino Acid Change	Genotypes (Sanger confirmed)	HGMD or dbSNP IDs	References
RD1-12	Sporadic	CRD	*C2orf71*	NM_001029883	c.1514G>A	p.W505*	Heterozygous	CM1511740	Yang et al [2015]
			C2orf71		c.3266dup	p.S1090Ifs*17	Heterozygous	Novel	
			BBS4	NM_033028.4	c.1375C>T	p.Q459*	Heterozygous	Novel	
			TYRP1	NM_000550.2	c.1557T>G	p.Y519*	Heterozygous	CM135790	Simeonov et al. [2013]
			SLC45A2	NM_016180	c.834C>G	p.Y278*	Heterozygous	CM083852	Hutton et al. [2008]
RD11-06	Sporadic	RP	*GUCY2D*	NM_000180.3	c.1724C>T	p.P575L	Heterozygous	CM023932/rs28743021	Small et al. [2008]
			TYR	NM_000372.4	c.1217C>T	p.P406L	Heterozygous	CM910385/rs104894313	Giebel et al. [1991]
			TYRP1	NM_000550.2	c.1261+1G>A	IVS9+1G>A	Heterozygous	rs140365820	
RD12-01	Sporadic	RP	*RP1L1*	NM_178857.5	c.416dup	p.G140Rfs*10	Heterozygous	rs201192645	
			TYR	NM_000372.4	c.721G>A	p.A241T	Heterozygous	CM145799 (DM?)	Fossbakk et al. [2014]
RD12-07	RP	Sporadic	*RHO*	NM_000539.3	c.936+1G>T	IVS4+1G>T	Heterozygous	CS920776	Hernan et al. [2011]
			TYRP1	NM_000550.2	c.1354A>G	p.M452V	Heterozygous	CM081465	Hutton et al. [2008]
			IMPDH1	NM_000883.3	c.931G>A	p.D311N	Heterozygous	CM020283	Bowne et al. [2002]
RD15-03	Sporadic	RP	*OCA2*	NM_000275.2	c.1004C>T	p.T335M	Heterozygous	rs533988694	
			TRPM1	NM_002420.5	c.1197G>A	p.P421=	Heterozygous	CS097758	Audo et al. [2009]
					c.215A>G	p.Y72C	Heterozygous	CM097760	Audo et al. [2009]
RD20-03	Sporadic	CSNB	*OCA2*	NM_000275.2	c.1513A>C	p.F505V	Heterozygous	None	
RD6-05	Sporadic	MD	*TIMP3*	NM_000362.4	c.29T>A	p.L10H	Heterozygous	Novel	predicted as pathogenic
			TYR_1	NM_000372.4	c.740G>T	p.C247F	Heterozygous	Novel	
RD11-07	Sporadic	RP	*OCA2*	NM_000275.2	c.1574C>T	p.P525L	Heterozygous	Novel	
RD11-08	Sporadic	RP	*IMPDH1*	NM_000883.3	c.931G>A	p.D311N	Heterozygous	CM020283	Bowne et al. [2002]
RD11-08			*TYRP1*	NM_000550.2	c.977G>A	p.R326H	Heterozygous	CM135784/rs16929374	
RD13-01	Autosomal dominant	CRD	*GUCA1A*	NM_000409.3	c.296A>G	p.Y99C	Heterozygous	CM980960	Payne et al. [1998]
			OCA2	NM_000275.2	c.40G>A	p.A14T	Heterozygous	rs368928996	
